# Preparation and Crystallization of Magnetic Glass‐Ceramics from the Residues after Sulfur Release and Iron Recovery from Copper Ore Tailings with Varied CaO Content

**DOI:** 10.1002/open.202100138

**Published:** 2021-10-04

**Authors:** Bing Luo, Tongjiang Peng, Hongjuan Sun, Tao Hui

**Affiliations:** ^1^ Key Laboratory of Solid Waste Treatment and Resource Recycle Ministry of Education Mianyang Sichuan 621010 P. R. China; ^2^ Analytical and Testing Center Southwest University of Science and Technology Mianyang Sichuan 621010 P. R. China; ^3^ Institute of Mineral Materials and Applications Southwest University of Science and Technology Mianyang Sichuan 621010 P. R. China; ^4^ City College Southwest University of Science and Technology Mianyang Sichuan 621000 P. R. China

**Keywords:** copper ore tailings, crystallization kinetics, direct sintering, magnetic glass-ceramics, residues

## Abstract

To comprehensively reuse copper ore tailings (COT), the fabrication of glass‐ceramics by the direct sintering method was investigated, where the residues after sulfur release and iron recovery from copper ore tailings were used as raw materials. The effect of the CaO added on the fabrication of glass‐ceramics was emphasized. After analysis of chemical composition and thermodynamics, crystallization kinetics were analyzed by Differential Scanning Calorimetry (DSC) and fitted to the Kissinger equation. The crystal phase and microstructure of sintered glass‐ceramics heated between 1080 °C and 1100 °C were estimated by X‐Ray diffraction analysis (XRD) and Scanning Electron Microscopy (SEM), respectively. Furthermore, the effects of the addition of CaO on the properties of the sintered glass‐ceramics have been discussed. The results showed that the magnetic glass‐ceramics were sintered by the residues successfully, the color of which was lighter than that of glass‐ceramics sintered by raw materials before iron recovery. According to the XRD analysis, hedenbergite, wollastonite and sekaninaite were formed with traces of maghemite and quartz. In terms of crystallization kinetics and sintering results, a decrease in the activation energies of crystallization and in sintering temperature were observed for an increase in the addition of CaO of up to 10 wt.%. Moreover, the properties of the sintered glass‐ceramics, including bulk density, linear shrinkage and flexural strength, were enhanced, while water absorption and true density were reduced with the increase of the amount of CaO added.

## Graphical abstract

**Magnetic glass‐ceramics** with good performance were fabricated successfully by direct sintering, using residues with a small amount of iron after sulfur release and iron recovery from copper ore tailings. Hedenbergite, wollastonite and sekaninaite were formed with traces of maghemite and quartz. Meanwhile, the color of the magnetic glass‐ceramics was lighter than that of glass‐ceramics sintered by raw materials before iron recovery.

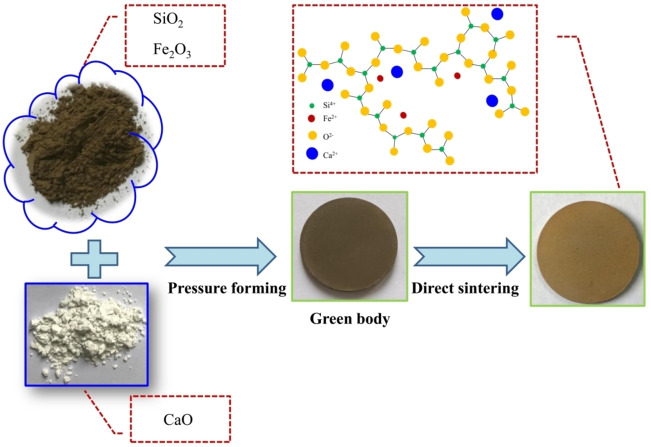

## 1 Introduction

Coper ore tailings (COT) are generated as the representative industrial bulk solid waste in the process of extracting target components from copper ores. It was estimated that approximately 400 t of COT were produced for 1 t of copper.[^1^, ^2^]

COT contain several valuable resources, such as sulfides (SO_x_), iron oxide, aluminum oxide, silicon oxide and other trace amounts of components. It has previously been suggested that COT could be recycled and thus utilized.[^1^, ^3^] With the rapid development of economic demand for mineral resources, the dramatic increase of the scale and intensity of exploitation and utilization for copper ores, over 36 million tons of COTS were produced worldwide and about 44.4 % were contributed by China each year.[^1^, ^4^] Large quantities of COT stacked in the natural environment could lead to various eco‐environmental problems. As large amounts of land resources have been seized, COT become a source of pollution, with the operating cost of enterprises increased. In addition, tailings dams are prone to security incidents.[^5^, ^6^, ^7^, ^8^] Thus, storing COT without any treatment of resource utilization could result in a serious waste of available resources. Therefore, from the perspective of resource utilization, it was essential to develop a highly efficient, scientifically evaluated and clean way of utilizing COT. This would not only eliminate the impact on the ecological environment, but also create economic benefits and promote the sustainable development of copper ores in the short and long term.

In order to actively respond to the call for comprehensive utilization of COT, a systematic approach for the successive release of sulfur and the recovery of iron by a linear route, including oxidation roasting and magnetic separation, were investigated in our previous study.^[2]^ We showed that sulfur could be released in a first step, followed by iron recovery. However, this process generates residues after sulfur release and iron recovery. The main components of the residues were SiO_2_, Al_2_O_3_, Fe_2_O_3_ and MgO, similar to the constituents needed for preparing glass‐ceramics.[^9^, ^10^]

Recently, extensive studies have been carried out on how to convert various tailings or residues from tailings as raw material in terms of their properties to value‐added glass‐ceramics with both glass phase and crystal phase by different heat treatments. It would be attractive for a better future of waste utilization both at home and abroad.[^11^, ^12^, ^13^] Since the early 1960s in Russia, many alternative ways have been developed to manufacture high‐value glass‐ceramics, including the melting‐sintering[^14^, ^15^, ^16^] and the sintering‐crystallization method.[^14^, ^17^] By melting‐sintering, a variety of ground materials were melted in a furnace, put into the mold and subsequently annealed.[^18^, ^19^] Using the sintering‐crystallization method, many types of materials, with additives, can be subjected to the series of melting, water quenching, grinding, pressing‐molding and heat treatment.^[20]^ No matter which method is chosen, a high temperature (more than 1300 °C) is needed for the raw materials to melt in the process of fabricating the parent glass, leading to relatively high energy consumption coupled with long durations and high costs. Thus, decreasing the processing temperature, reducing the production costs and shortening the route became the first considerations in the preparation of glass‐ceramics. In view of this, the direct sintering by one‐step process was conducted to prepare the desired glass‐ceramics, similar to the production of ceramics.^[21]^ According to this method, the raw material is ground, press‐ molded into a green body, crystallized and sintered to obtain glass‐ceramics with good properties.^[22]^ However, if the content of iron in the raw material exceeds 40 %, it poses a barrier to the preparation of glass‐ceramics.^[23]^ The high iron content of material could lead to serious overflow in the smelting process and a darkening in color. This led to a low application value of iron‐rich glass‐ceramics and, deeming them unsuitable for architectural decoration.[^18^, ^24^] Accordingly, only a few studies were concerned with the fabricating of glass‐ceramics using COT. To this point, our previous study showed that the iron of the COT could be recycled with a yield rate of 67.21 %, leading to a low iron content. Therefore, the residues with small portion of the same composition as glass‐ceramics were deemed suitable to prepare glass‐ceramics.

In this study, the main purpose was to maximize the use of the residues to prepare glass‐ceramics using direct sintering and promote the performance by adding different amounts of CaO to decrease the sintering temperature in different heat treatments. Furthermore, the crystallization kinetics estimated by Differential Scanning Calorimetry (DSC) at different amounts of added CaO should be obtained. Furthermore, the effect on physical and magnetic properties of the sintered glass‐ceramics from adding CaO was to be subsequently investigated, all in all hoping to disclose an environmentally friendly – clean, safe and sustainable – yet economic approach to using COT as raw materials.

## Experimental Section

### Materials Preparation and Devices

Residues collected from a previous study have been used as raw material in this study.^[2]^ The residues were produced successively through a series of treatments including oxidizing roasting, sulfur release and magnetic separation of the copper ore tailings in optimum conditions. The residues as samples were grounded into powders below 200 meshes and dried at 105±2 °C for 24 h. The main chemical components with more than 0.4 wt.% are shown in Table 1. Based on the chemical composition of the samples, the samples contained 49.71 wt.% SiO_2_, 9.61 wt.% Al_2_O_3_, 6.36 wt.% CaO, 4.83 wt.% K_2_O, 4.1 wt.% Na_2_O, respectively, which are the main silicon, aluminum, calcium, potassium and sodium source for glass‐ceramics.

**Table 1:** Composition of formula used in preparation of glass‐ceramics [wt.%].

Sample	Residue	Add	CaO	SiO_2_	Fe_2_O_3_	Al_2_O_3_	MgO	K_2_O	Na_2_O	ZnO	CuO	Other
BZ‐0 wt.%	100 %	0 %	6.36	49.74	11.07	9.61	7.80	4.83	4.1	3.12	2.45	0.86
BZ‐5 wt.%	95 %	5 %	11.04	47.25	10.52	9.13	7.41	4.59	3.90	3.02	2.33	0.82
BZ‐10 wt.%	90 %	10 %	15.72	44.77	9.96	8.65	7.02	4.35	3.69	2.86	2.21	0.77

**Figure 1:**
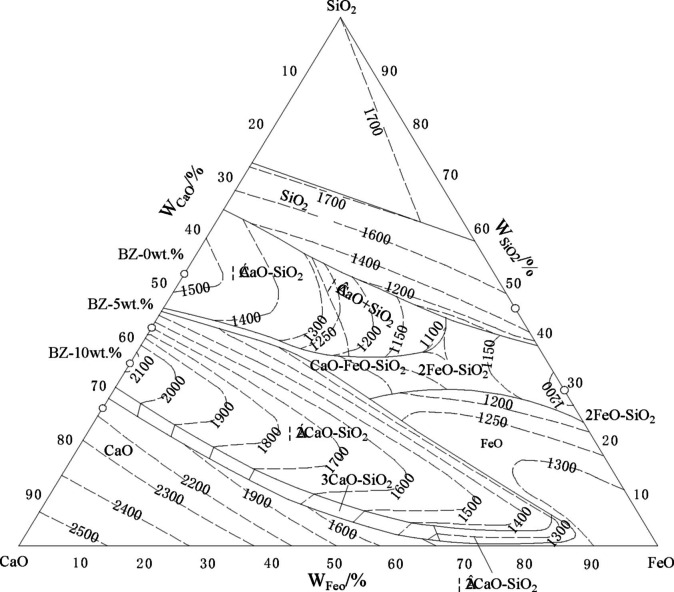
Phase diagram of CaO‐FeO‐SiO_2_.

Based on the CaO−FeO−SiO_2_ phase diagram^[25]^ shown in Figure 1, three groups of formulations were designed and labelled as BZ‐0 wt.%, BZ‐5 wt.% and BZ‐10 wt.%. The ingredients of the batches are shown in Table 1, showing the variation in residue content upon setting the CaO content to 0 %, 5 % and 10 %, respectively.

Calcium oxide and polyvinyl alcohol (PVA) were both analytically pure, and were bought from Sinopharm Chemical Reagents Co. Ltd, China. The main instruments were listed as follows: XMQ‐Φ240*90 conical ball mill (Wuhan Prospecting Machinery Factory, China), YP‐20tb oil pressure powder tableting machine (Hebi Lixin Instrument & Meter Co Ltd, China) and QSH‐ABF‐1700T‐1515 box atmosphere furnace (Shanghai Quanshuo Electric Stove, Co Ltd, China).

### Experimental Procedures

**Figure 2:**
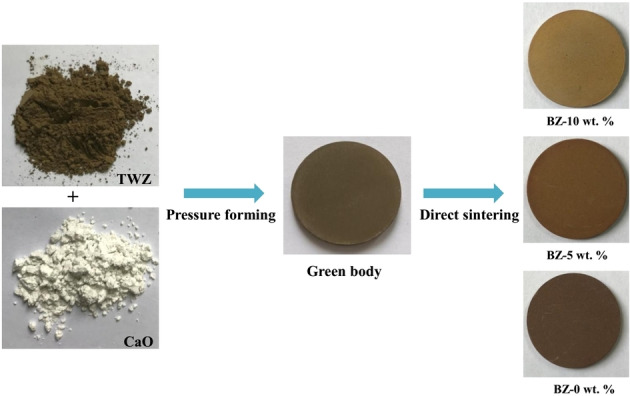
Experimental process of the preparation of glass‐ceramics with varied addition of CaO.

The green bodies were prepared by using 3 g mixtures of different batches, respectively, with 5 wt.% PVA aqueous solutions to gain a high‐strength green body, in which the ratio of liquid to solid was 19 : 1. Then, the adhesive powder was pressed into a green body (25 mm diameter, 4–6 mm thickness) using the oil pressure powder tableting machine at 22 MPa. The green body was first heated from room temperature to 1000 °C with a heating rate of 10 °C min^−1^, and then further heated to 1080 °C, 1085 °C, 1090 °C, 1095 °C and 1100 °C respectively with a heating rate of 5 °C min^−1^ for a holding time ranging from 10 min to 60 min, to obtain glass‐ceramics. Subsequently, the sintered glass‐ceramics, denoted by BZ‐0, 5 or 10 wt.% and sintering temperature‐holding time, were cooled to room temperature. The schematic diagram of the preparation of glass‐ceramics is presented in Figure 2.

### Analytical Methods

The crystal phase of both the residue and sintered glass‐ceramics was confirmed by X‐ray diffraction (XRD, X′Pert pro, step of 0.02° in 2θ ranging from 3° to 80°) using Cu Kα radiation(λ=0.15406 nm). The chemical composition of the residues was investigated by X‐ray fluorescence spectroscopy (XRF‐Axios, 2.4 Kw, Netherlands, content range 0.01 %–100 %, sample diameter 32 mm, θ/2θ scanning mode of the goniometer) using a Rh target according to JY/T 016–1996. The micromorphology of the sintered glass‐ceramics was characterized by scanning electron microscopy (SEM‐EDS, Ultra 55, 15 kV) with magnifying multiple ranges from 3000× to 200×. The magnetic properties of sintered glass‐ceramics were measured by a vibrating sample magnetometer (VSM BKT‐4500Z, China). The crystallization peak temperature (T_f_) of residues with different CaO content were determined by a thermal analyzer (DSC apparatus, Netzsch STA 449F5), operated at different heating rates of 5, 10 and 15 °C min^−1^ from room temperature to 1200 °C in air atmosphere. Simultaneously, HSC Chemistry Software 6.0 was employed to carry out basic thermodynamic calculations.

Water absorption, linear shrinkage, bulk density, true density, open porosity and closed porosity were determined using standard tests according to GB/T 9966.3‐2001. All of the properties of sintered glass‐ceramics were measured in triplicate, and the average of three results is reported.

## 2 Results and Discussion

### 2.1 Theoretical and Thermodynamic Analysis

**Figure 3:**
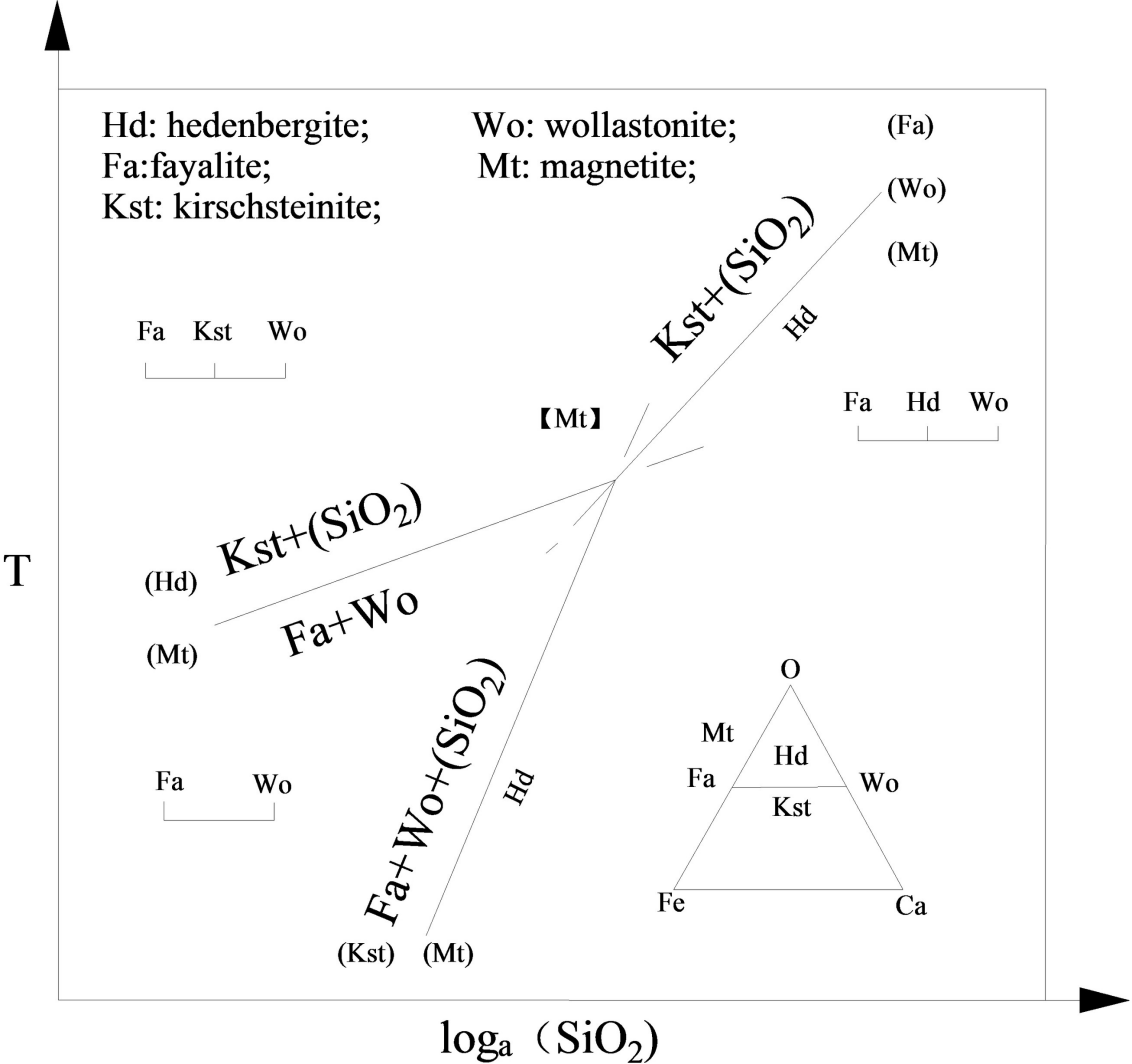
Effect of temperature and SiO_2_ on the products of Equations (1) and (3).

As can be seen from Table 1, various oxides were predominant in raw materials and formulations, especially for SiO_2_. Some components could react with each other in the solid state to form new compounds, corresponding to the following reactions in Equations (1)–(3). According to these reactions, the effects of temperature and SiO_2_ on the products is depicted in Figure 3.^[26]^ An increase of SiO_2_ content is beneficial to the generation of hedenbergite (Hd), and magnetite (Mt) can stably coexist with all the bivariant combinations in the Fayalite‐Kirschsteinite‐Magnetite‐Hedenbergite system. On the macro level, the sintering process was carried out under oxidizing atmosphere. But, as near the surface of combustion particles the concentration of CO is very high, a local reducing atmosphere also exists, allowing Fe_3_O_4_ to be converted into FeO by CO above 900 °C.

(1) $$CaO+{SiO}_{2}\to Ca{SiO}_{3}$$

(2) $${Fe}_{3}O_{4}+CO↑\to 3FeO+{CO}_{2}$$

(3) $$CaO+FeO+2{SiO}_{2}\to CaFe{(Si}_{2}O_{6})$$

**Figure 4:**
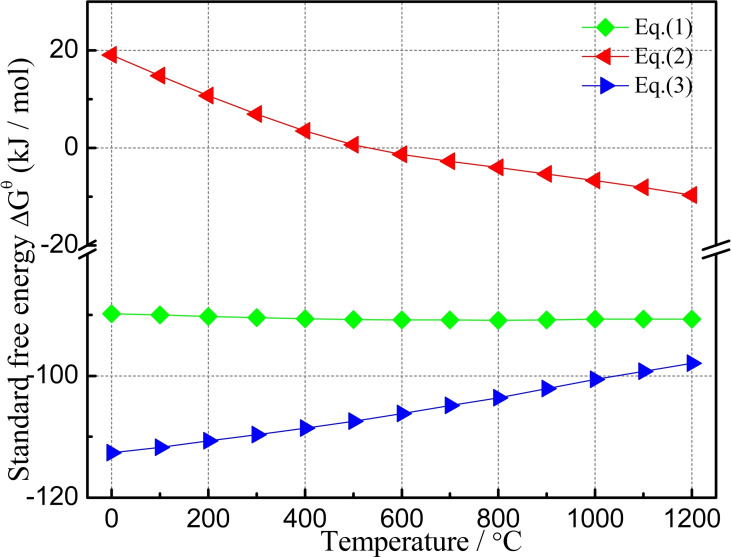
Standard free energy changes for reactions vs. temperature for Equations (1)–(3).

The variation of standard free energy (▵G^θ^) with respect to temperature for reactions (1)–(3) is depicted in Figure 4. ▵G^θ^ for Equations (1) and (3) is negative from room temperature to 1200 °C, indicating that the formation of wollastonite and hedenbergite is thermodynamically possible. Reaction (2) becomes possible with increasing temperature in the range of 500 °C‐1200 °C.

### 2.2 Crystallization Kinetics of Sintered Glass‐Ceramics

**Figure 5:**
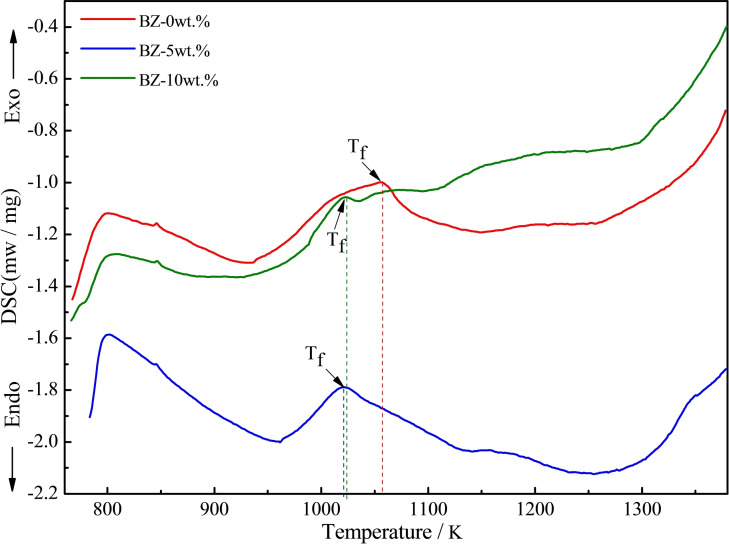
DSC of samples with varied addition of CaO (α=5 °C ⋅ min^−1^).

Figure 5 shows the heat effect of the glass samples with a heating rate of 5 °C min^−1^ through their DSC curves. The exothermic peak corresponds to the release of latent heat of crystallization of the glass samples to generate entropy change, which would affect or even hinder the sintering process.^[27]^ With increasing content of CaO, the exothermic peak turns to reduced temperatures, indicating a decrease in crystallization temperature (T_f_). In addition, the exothermic peak becomes sharper, implying an increase in crystallization rate. This could be due to the synergistic action of FeO, Fe_2_O_3_, SiO_2_ and CaO. SiO_2_ is a typical network generator because of the complex structure silicate formed by many [Si−O] covalent bonds.^[25]^ FeO and Fe_2_O_3_ both act as network modifier in the glass, thus potentially playing a key role in breaking [Si−O−Si] bonds and increasing the content of non‐bridging [Si−O−Fe] oxygen bonds, result in a weakening of the glass network.^[16]^ This indicates that network depolymerization was promoted and small crystal particles were formed in the glass phase. As a typical network modifier, CaO could also destroy the polymerization of silicon oxides by breaking [Si−O−Si] bonds and creating the non‐bridging [Si−O−Ca] oxygen bonds. So, the formation and separation of crystal particles from the glass phase could accelerate the crystallization of the samples.^[26]^ Once the glass network structure has been broken, some ion channels are generated. Ca^2+^ and Fe^2+^ travel to the outermost layer of the crystal and promot the continuous growth of the Hedenbergite (CaFeSi_2_O_6_) and Wollastonite (CaSiO_3_) phases.^[28]^

In order to further confirm the transformation of crystallization behavior with the influence of CaO content detailed above, non‐isothermal DSC was carried out to analyze the crystallization kinetics. The exothermic peak temperatures of samples with different CaO content at various heating rates ranging from 5 °C min^−1^ to 15 °C min^−1^ are depicted in Table 2. Under non‐isothermal conditions, the Kissinger equation [Eq (4)] and the Augis‐Bennett equation [Eq (5)] were employed to derive the crystallization activation energy (E_c_) and crystal growth index (n), respectively.

(4) $$\ln\left(\frac{T_{f}^{2}}{\alpha }\right)=\frac{E_{c}}{RT_{f}}+\ln\left(\frac{E_{c}}{T_{f}}\right)-ln\left(v\right)$$

(5) $$n=\frac{2.5RT_{f}^{2}}{\Delta {TE}_{c}}$$

**Table 2:** Crystallization kinetics analysis parameter of the three samples.

sample	T_f_ [K]			E_c_ [kJ ⋅ mol^−1^]	n (α=5 °C ⋅ min^−1^)
	α=5 °C ⋅ min^−1^	α=10 °C ⋅ min^−1^	α=15 °C ⋅ min^−1^		
BZ‐0 wt.%	1057	1071	1082	402.44	2.24
BZ‐5 wt.%	1029	1046	1055	361.82	2.68
BZ‐10 wt.%	1026	1045	1053	340.85	2.94

**Figure 6:**
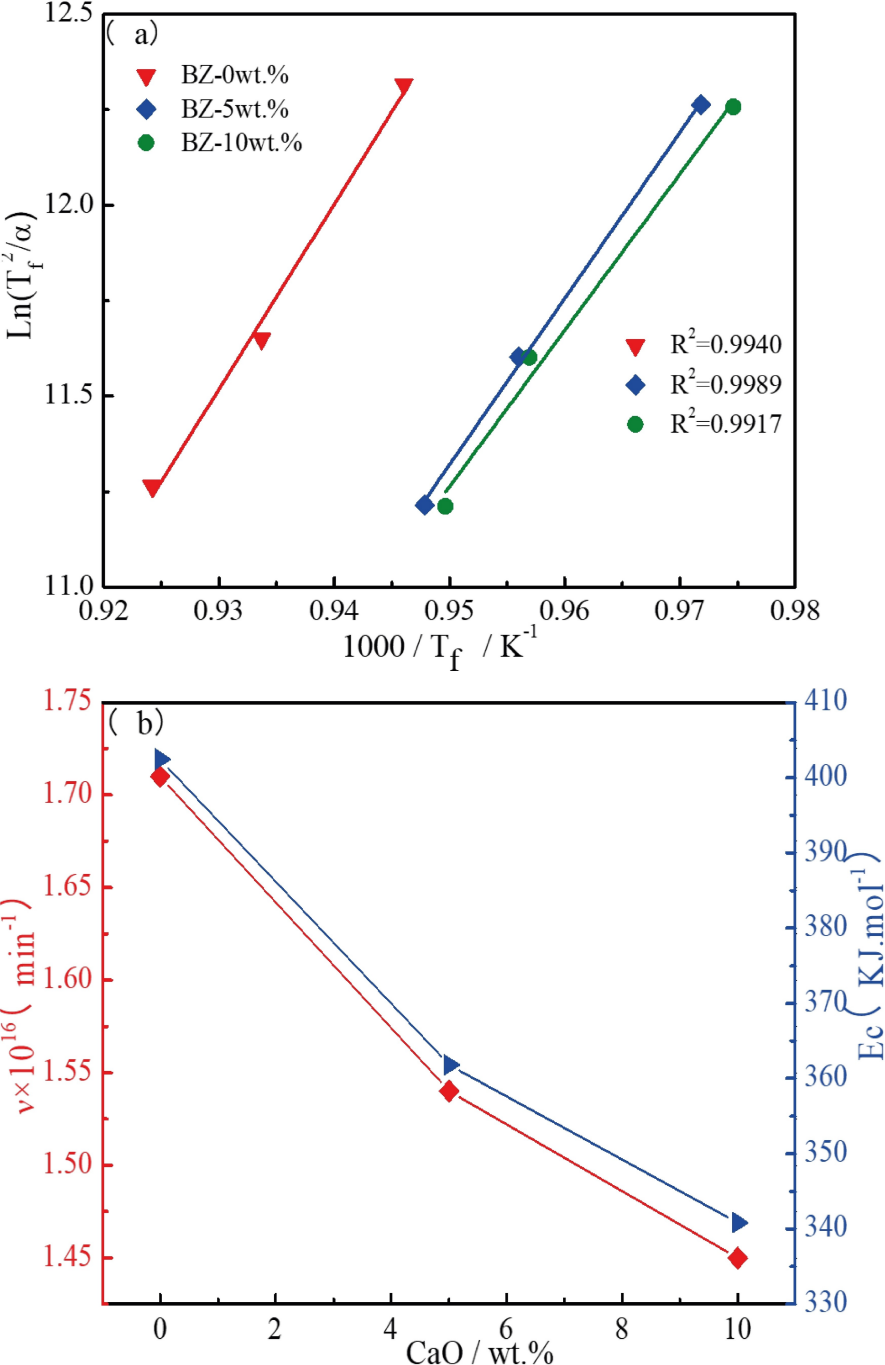
The crystallization kinetics of samples: (a) Kissinger curve of samples; (b) variation of crystallization activation energy and frequency factor.

T_f_ is the temperature of the crystallization exothermal peak taken from the DSC curve. E_c_ denotes the crystallization activation energy, α is the heating rate and v is the frequency factor. R is the universal gas constant (8.314 J mol^−1^ K^−1^), n denotes the crystal growth index and ▵T is the full width at half maximum intensity of the exothermic peak. In accordance with Equation (4), a linear relationship between ln (T_f_^2^/α) and 1/T_f_ was established in Figure 6(a), where the slope yield value (E_c_/R) and the intercept (ln(E_c_/E_f_)‐ln(v)) of the linear fit were obtained, respectively. E_c_ and v were further calculated as shown in Figure 6(b). According to Equation (5), n was evaluated with respect to the crystal growth mechanism. Eventually, E_c_, v and n with various CaO content at different heating rate could be evaluated as shown in Table 2.

E_c_ values ranging from 340.85 to 402.44 kJ mol^−1^ and v values in the range of 1.45×10^16^–1.71×10^16^ min^−1^ are shown in Figure 6(b). With the increase of CaO content, the activation energy and frequency factor of crystallization decreased gradually, indicating that the crystal evolution ability of samples increases gradually, and the crystal growth rate decreases gradually. The increase of CaO content enhanced the amount of free oxygen in glass, thus breaking the silica tetrahedra,^[27]^ resulting in a looser glass structure which was beneficial to ion migration and accelerated the viscous flow and mass transfer process. Because of this, a lower temperature for the crystallization and sinter of glass‐ceramics was determined with the appropriate addition of CaO: as a glass network outer body oxide, CaO can weaken the glass network structure, reducing the degree of network polymerization, thus speeding up the iron diffusion to promote crystallization,^[25]^ in turn leading to precipitation of hedenbergite and wollastonite. In addition, less crystallization activation energy was needed to break the non‐bridging oxygen network structure.^[29]^ However, due to the CaO‐promoted weakening of [Si−O−Si] bonds, the content of CaO must not be too high to prevent short or fast melting properties. This would easily increase the risk of specimen deformation.^[30]^ Accordingly, in this study, the added CaO content could not exceed 10 wt.%, otherwise, the green body would show cracks.

The crystal growth index (n), also called the Avrami parameter, is listed in Table 2, the values of which were calculated according to Equation (5). In this study, n was performed with a heating rate of 5 °C min^−1^ from 0 wt.% to 10 wt.%. It could be seen that the n value of these three samples with the increasing CaO content were determined as 2.24, 2.68, 2.94, respectively, implying that two‐dimensional growth of glass played a key role in the surface crystallization mechanism.^[30]^

### 2.3 Phase Composition of Sintered Glass‐Ceramics

**Figure 7:**
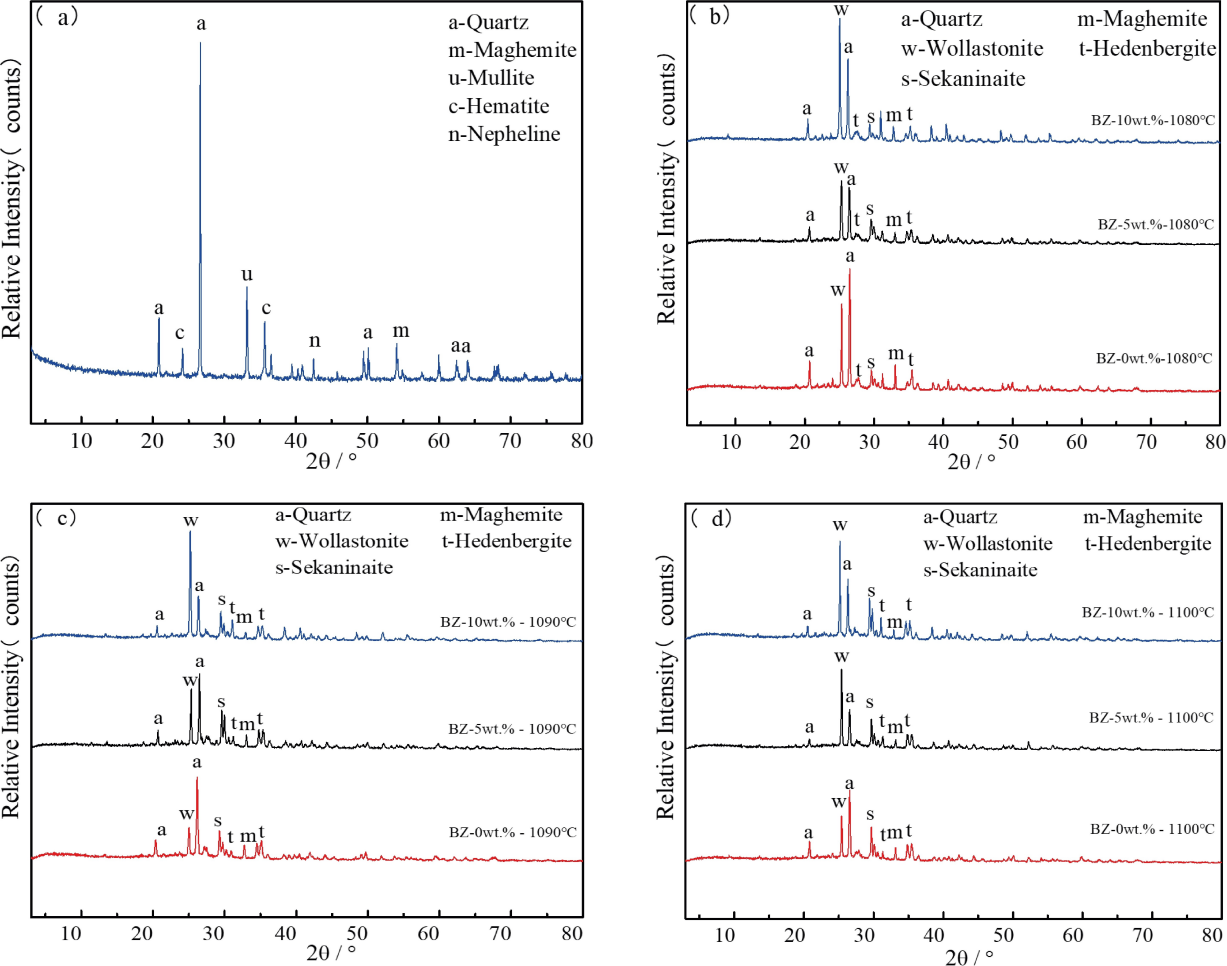
XRD spectra of (a) residues after sulfur release and iron recovery, (b)–(d) varied CaO content at 1080 °C, 1090 °C and 1100 °C for 30 min, respectively.

As illustrated in Figure 7(a), the phase compositions of residues mainly consisted of quartz, maghemite, hematite, a small amount of mullite and nepheline. These played a key role in the preparation of glass‐ceramics as well as non‐bridging oxygen bonds [Si−O−Fe] created in the sintering process.^[31]^ In order to investigate the sintering behavior influenced by the temperature and CaO content in crystal growth process, it was necessary to determine the various crystalline phases generated in glass‐ceramics at different temperatures with various CaO contents,^[25]^ as these could affect the physical and chemical properties of the glass‐ceramics. The XRD patterns of the glass‐ceramics at various temperatures and CaO contents are presented in Figures 7(b)–(d). It was evident that, after sintering treatment at 1080 °C, 1090 °C and 1100 °C, new phases of every sintered glass‐ceramic with varied CaO content was formed including wollastonite, hedenbergite and sekaninaite, with traces of maghemite and quartz, underlining the above‐mentioned reactions (1)–(3) between the different components

Although the glass‐ceramics were obtained by direct sintering process and thus not a controlled crystallization process, hence lacking the typical vitrification, the newly created phases were quite special for ferromagnetic glass‐ceramics belonging to the FeO−Fe_2_O_3_−CaO−SiO_2_ system. They could be considered as an effective thermal seed material (microcrystalline glass) with biological activity and magnetism for the treatment of cancer in thermotherapy.^[26]^ Mixtures with a high SiO_2_ content could combine FeO and CaO to form low melting point calcium and iron silicate compounds.^[5]^

In fact, FeO was a good nucleating agent and Fe^2+^ as network interstitial ions could also break the [Si−O] net structure,^[32]^ gradually reducing the viscosity of glass and promoting the crystallization of hedenbergite in turn. The hedenbergite could be used as a fortifier to decrease the viscosity of glass in the body and easily wet SiO_2_ particles and rapidly promoted the sintering of the body.^[8]^ At the same time, Ca^2+^ played the same role as Fe^2+^ as both are considered as network interstitial ions in the glass phase, which decreased particle migration resistance and promoted the phase separation and crystallization of glass.^[30]^ With the addition of CaO, wollastonite was formed. When the temperature exceeded 1100 °C, the glass‐ceramics with 5 wt.% and 10 wt.% CaO content began to bubble. The glass‐ceramics with 0 wt.% CaO were sintered above 1250 °C. When the sintering temperature was lower (1080 °C), it was beneficial to the crystallization process.

**Figure 8:**
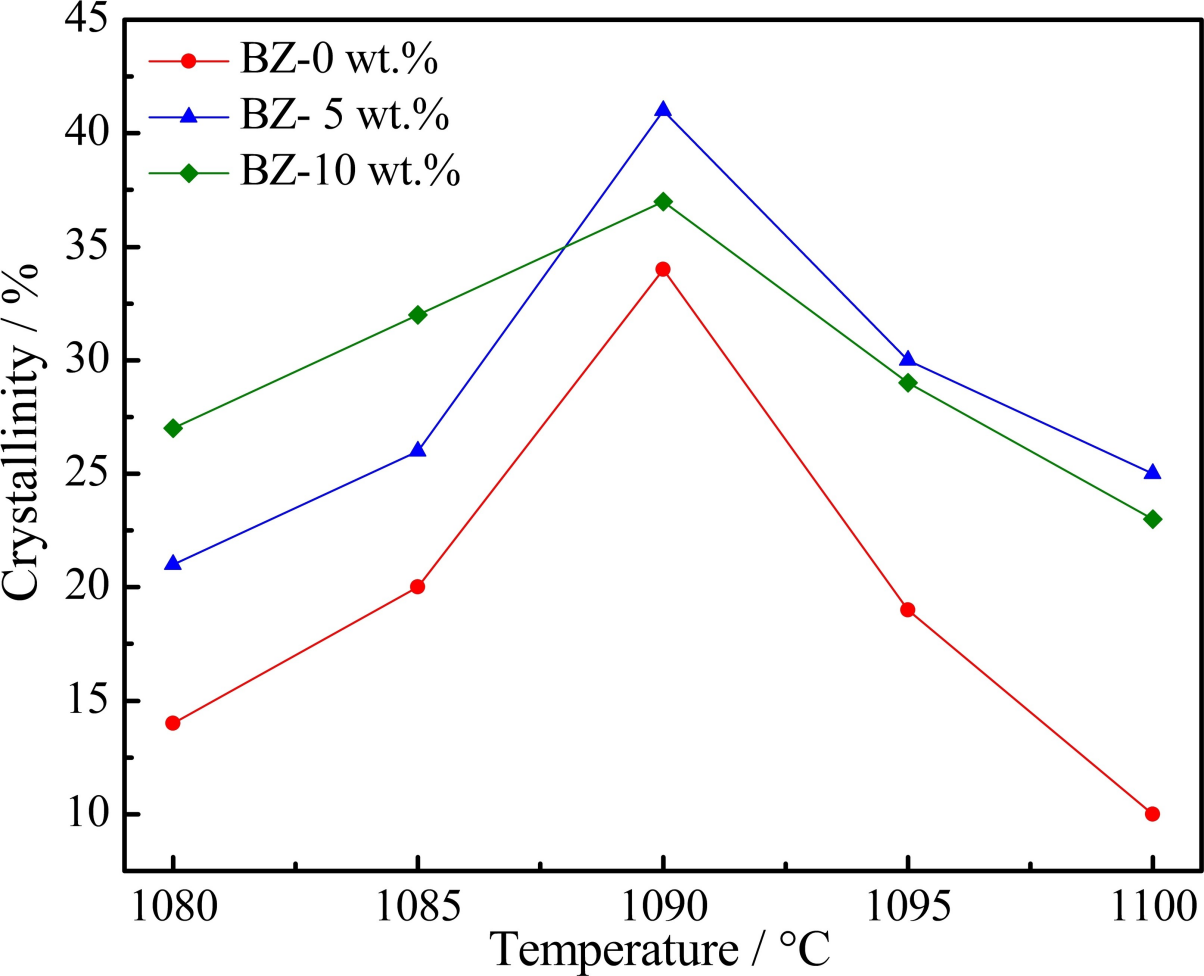
Crystallization degree of glass‐ceramic at different temperature

However, the crystallinity of sintered glass‐ceramics decreased with increasing temperature for all samples, as illustrated in Figure 8. An equilibrium between crystallization and the sintering process was achieved at 1090 °C; due to better conditions for crystal precipitation, a maximum degree of crystallization was achieved. As the viscosity of glass decreased due to the addition of CaO, the glass particle interface binds faster. This could result in a relatively small amount of interface for crystal growth and a gradual decrease in the degree of crystallization.^[27]^ As a result, the final sintering temperature was determined to be 1100 °C with the addition of CaO from 5 wt.% to 10 wt.%.

### 2.4 Microstructure of Sintered Glass‐Ceramics

**Figure 9:**
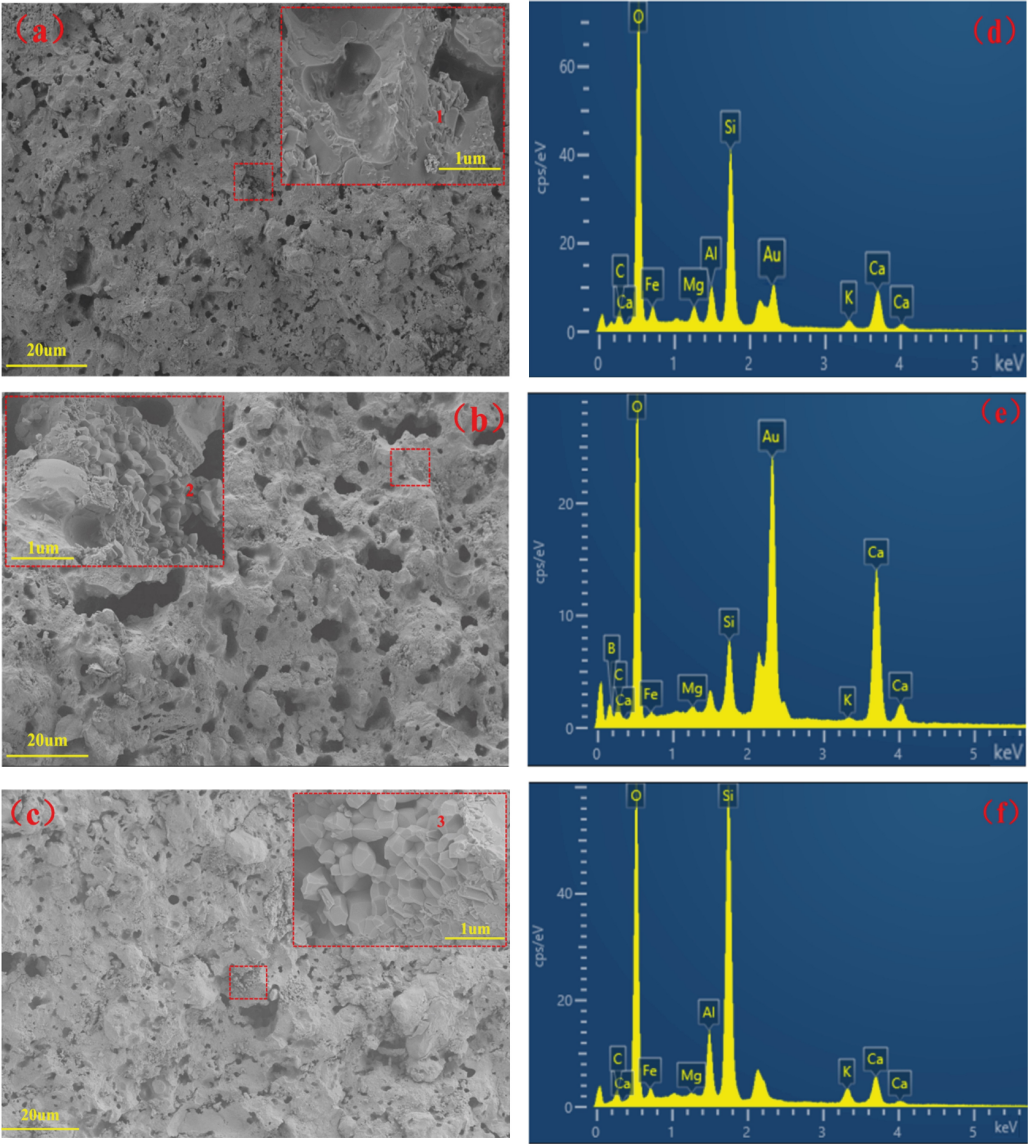
SEM images of glass‐ceramic samples (fracture surface) at 1100 °C for 30 min (a, b, c: The microstructure of BZ‐0 wt.%, BZ‐5 wt. % and BZ‐10 wt.%, respectively; d, e, f: the composition of 1, 2 and 3 in a, b, and c, respectively).

Figure 9 shows the SEM‐EDS images of glass‐ceramics sintered at 1100 °C for 30 min with the addition of 0, 5 and 10 wt.% CaO in fractured surfaces‐ By comparing Figures 9(a), 9(b) and 9(c), it can be clearly seen that the samples the degree of sintering increased alongside the addition of CaO, which was in accordance with the changed pore size as a result of the densification from the sintering process. At the same time, most of the pores presented were closed. As clearly shown in the highlighted red area in Figures 9(a), 9(b) and 9(c), partial crystallization was confirmed, illustrated by crystals formed in nonporous regions. With the increase of CaO content, the pore size became smaller and more and more regularly‐shaped crystals grew. As crystal phase formation increased, the glass viscosity also increased, leading to lower sintering rates or even the termination of sintering.^[33]^ As can be seen from Figure 9, the crystalline phases (point 1, 2 and 3) mainly contain Fe, Si, O and Ca, consistent with the XRD results discussed above. Two mineral phases, including wollastonite and hedenbergite, constituted a network structure and interpenetrated with each other,^[5]^ suggesting that it was conducive to the penetration of the glaze layer in the future

### 2.5 Physical and Magnetic Properties of Sintered Glass‐Ceramic

**Figure 10:**
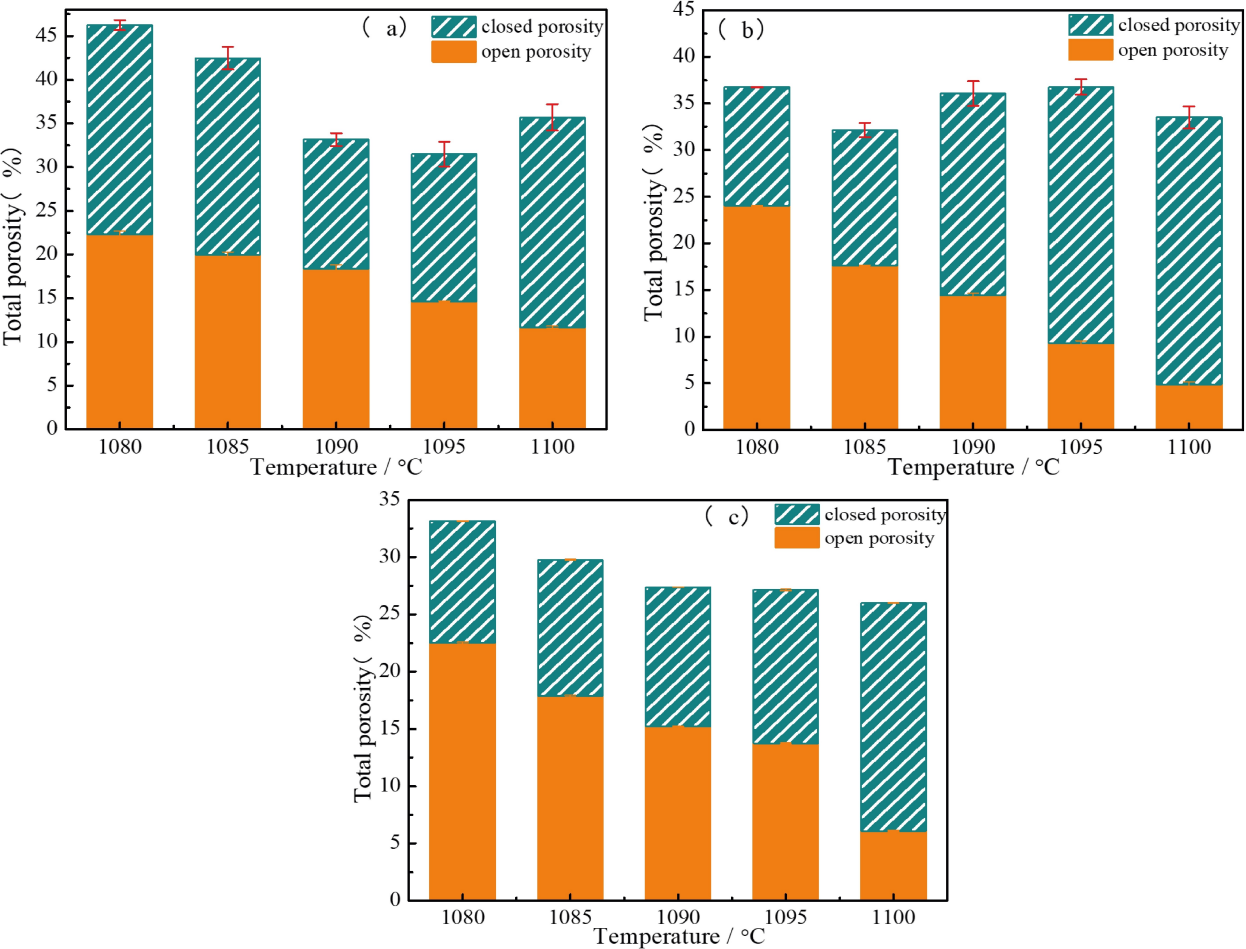
Porosity of sintered glass‐ceramics (a) BZ‐0 wt.%, (b) BZ‐5 wt.%, (c) BZ‐10 wt.%.

**Figure 11:**
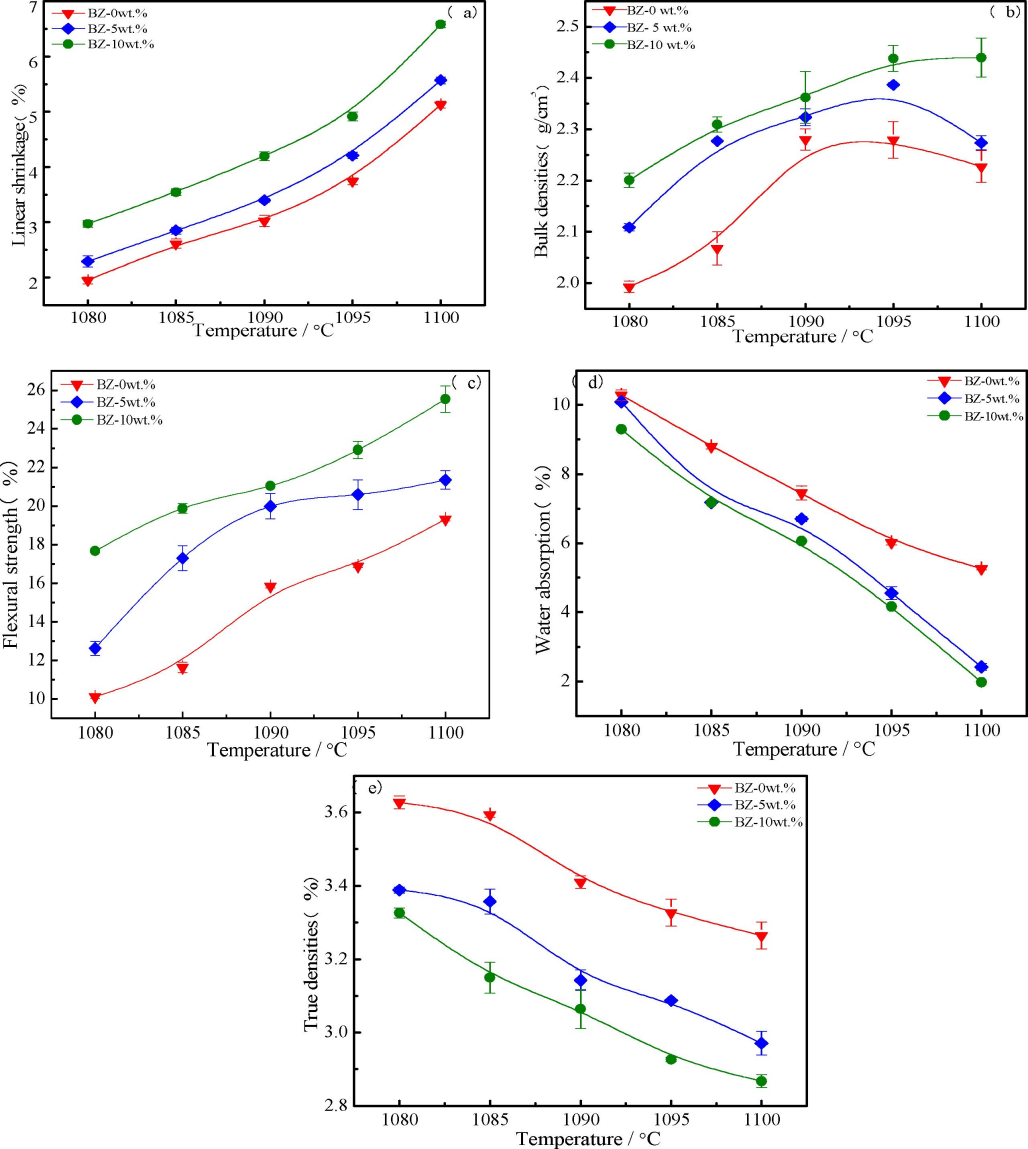
Properties of samples: (a) linear shrinkage,(b) bulk density, (c) flexural strength, (d) water absorption, (e) true density.

As discussed above, all sintered samples with varied CaO content at different temperatures contained crystalline and amorphous phases, forming multi‐phase composite structures, which led to the dual properties of glass and ceramics. Figure 10 and Figure 11 display the physical properties of all sintered samples with the increase in CaO content and sintering temperature for 30 min. In terms of each component of CaO added alone as shown in Figure 10(a), (b) or (c), with increasing temperature, the degree of open porosity gradually decreased while the closed porosity increased continually, implying that open pores turned to closed pores in accordance with typical sintering process.^[34]^

From Figure 11, it can be seen that water absorption is related to the degree of closed porosity: the more closed pores, the lower the water absorption. The water absorption of all sintered glass‐ceramics decreased sharply with the increase of sintering temperature. Samples generated with the addition of 10 wt.% CaO had lower water absorption at higher temperatures. Density measurements were performed and showed the opposite tendency to water absorption with the increase temperature and addition of CaO content.^[30]^ As the addition of CaO content increased from 0 wt.% to 10 wt.%, the density increased from 1.99 g cm^−3^ to 2.44 g cm^−3^. In addition, lower densities correlated with smaller flexural strength. With increasing CaO content, the flexural strength increased. The maximum flexural strength of sintered the samples was determined to 25.53 MPa at 1100 °C for 30 min with 10 wt.% CaO. Obviously, at the same temperature, with the increase of CaO content, the line shrinkage, bulk densities and flexural strength of all samples gradually increased while the water absorption and true densities gradually decreased. The sintering shrinkage of samples with the addition of 5 wt.% and 10 wt.% CaO was mainly completed from 1080 °C to 1090 °C, and almost no shrinkage occurred above 1095 °C. This was related to the difference of glass viscosity caused by the amount of crystallization and the proportion of glass phase in glass‐ceramics.^[35]^ At the same time, the linear shrinkage of samples without the addition of CaO continued to increase because the sample had not yet been fully sintered, indicating that the sintering temperature could be reduced with appropriate addition of CaO as fusing agent.

**Figure 12:**
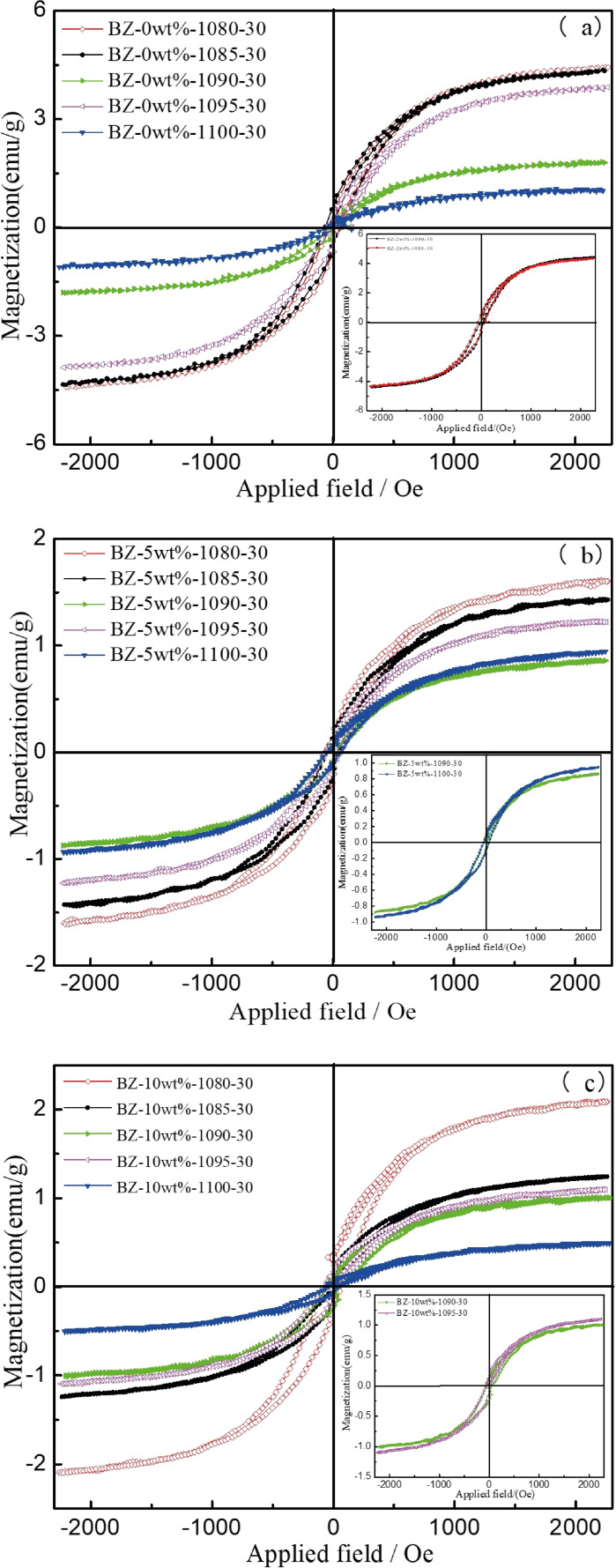
Magnetization curve of sintered glass‐ceramic at different temperature for 30 min.

Figure 12 depicts the hysteresis curves for sintered samples with the varied addition of CaO at different sintering temperatures. Through comparison, it was noticed that no matter the amount of CaO, the saturation magnetization (M_s_) of samples decreased with the increase of temperature, as expected, for samples sintered at 1090 °C and 1095 °C. With increasing CaO content, the distance of hysteresis curves and thus M_s_ of the sintered glass‐ceramics sintered at 1090 °C and 1095 °C became ever smaller. Moreover, with an higher amount of CaO, the M_s_ at the same temperature decreased gradually.

As a whole, the change of the M_s_ could be attributed to the reduction of magnetic crystals produced in the glass‐ceramic samples. With an increasing amount of CaO, the Ca^2+^ cations with their larger radius tend to enter the larger instead of the Fe^3+^ cations normally localized there.[^18^, ^23^] This leads to the decrease of difference in magnetic moments of atoms between B‐site and A‐site. In addition, the formation of wollastonite could also affect the distribution of magnetic particles.^[30]^ Hence, the M_s_ was reduced. The minimum M_s_ at 1100 °C with the addition of 10 wt.% CaO was 0.49 emu g^−1^. In general, the magnetic glass‐ceramic prepared in this study was more or less similar to ferromagnetic glass‐ceramics used as good heat seed material for thermotherapy.

## 3 Conclusion

In this study, the residues after sulfur release and iron recovery from copper ore tailings were used as raw material to successfully fabricate glass‐ceramics by direct sintering. The main conclusions were drawn as follows:

1. With varied addition of CaO at different sintering temperature for 30 min, the main crystal phases in glass‐ceramics were wollastonite, hedenbergite and sekaninaite, with traces of maghemite and quartz.
2. The addition of CaO not only reduced the activation energy of crystallization by weakening the interface irregularity between glass and crystal, but also decreased the frequency factor by increasing the viscosity of glass. Moreover, the sintering temperature for optimum densification of glass‐ceramics was found to be 1100 °C, which also decreased with the amount of CaO to up to 10 wt.%. Compared with the molten sintering process, it was possible to make the process less cumbersome and to reduce the energy consumption in preparing glass‐ceramics by the direct sintering method.
3. According to the results, at 1100 °C for 30 min, a higher amount of CaO led to greater bulk density. The stronger the flexural strength, the smaller the water absorption, and the lower the true density was. At the same time, the line shrinkage showed no significant changes with the addition of CaO from 5 wt.% to 10 wt.%. Besides, almost all sintered glass‐ceramics were magnetic, which suggests that they could be used as thermal seed materials in cancer thermotherapy.
4. The feasibility of the approach for preparing glass‐ceramics using COT residues by direct sintering was demonstrated, implying that further use of the residues was possible. Combined with our previous study in sulfur release and the recovery of γ‐Fe_2_O_3_, the comprehensive utilization of copper ore tailings was meaningful and feasible, which not only promoted zero‐emission of copper ore tailings to reduce the environmental impact environment, but also generated certain economic benefits with the recovery of sulfur, γ‐Fe_2_O_3_ and the production of glass‐ceramics, successively.

## Conflict of interest

The authors declare no conflict of interest.
